# Caregivers’ Implementation of Home Modifications for Older Care Recipients

**DOI:** 10.1093/geroni/igaf122.2822

**Published:** 2025-12-31

**Authors:** Mengzhao Yan, Kristen Glennie, Daniel Barbakoff, Bernard Steinman

**Affiliations:** University of Southern California, Los Angeles, California, United States; Wyoming Institute for Disabilities, Laramie, Wyoming, United States; Weill Cornell Medicine, New York, New York, United States; University of Wyoming, Laramie, Wyoming, United States

## Abstract

Home modifications can make caregiving tasks easier by enhancing safety and accessibility, yet it is not clear which factors are associated with the implementation of home modifications by caregivers on behalf of those receiving care. This study analyzed linked data from Round 12 of the National Health and Aging Trends Study and the National Study of Caregiving. Guided by the Ecology Theory of Aging and Anderson’s Behavioral Model, we conducted multivariate logistic regressions to explore whether sociodemographic characteristics, the care recipients’ health status (measured by the ICF framework), and caregiving activities are associated with family caregivers’ actions to implement home modifications. Key findings include: (1) Compared to spousal caregivers, adult children (OR: 0.48, p < 0.001) and other caregivers (OR: 0.27, p < 0.001) were less likely to make home modifications for care recipients; (2) More restrictions in social participation (OR: 1.14, p < 0.05) and more physical impairments (OR: 1.30, p < 0.001) among care recipients were associated with higher odds of making home modifications by caregivers; (3) Caregivers’ frequent assistance with self-care (OR: 2.18, p < 0.001), caregivers’ receiving training (OR: 1.78, p < 0.001), and care recipients’ use of rehabilitation (OR: 1.54, p < 0.001) were associated with higher odds of caregivers making home modifications. The findings highlight that increased caregiver responsibility and training play an important role in home modification implementation. Future research, policy, and practice to promote home modifications for aging in place should take caregivers’ experiences and activities into account.

